# Impact of Maternal Death on Household Economy in Rural China: A Prospective Path Analysis

**DOI:** 10.1371/journal.pone.0134756

**Published:** 2015-08-06

**Authors:** Fang Ye, Deng Ao, Yao Feng, Lin Wang, Jie Chen, Dale Huntington, Haijun Wang, Yan Wang, Economic Impact of Maternal Deaths in China

**Affiliations:** 1 Department of Preventive Health Care, China-Japan Friendship Hospital, Beijing, China; 2 Division of Maternal and Child Health, School of Public Health, Peking University, No.38 Xueyuan Road Haidian District, Beijing, China; 3 Asia Pacific Observatory on Health Systems and Policies, World Health Organization Western Pacific Regional Office, Manila, Philippines; 4 Institute of Child and Adolescent Health, School of Public Health, Peking University, No.38 Xueyuan Road Haidian District, Beijing, China; National Institute for Viral Disease Control and Prevention, CDC, China, CHINA

## Abstract

**Objectives:**

The present study aimed to explore the inter-relationships among maternal death, household economic status after the event, and potential influencing factors.

**Methods:**

We conducted a prospective cohort study of households that had experienced maternal death (n = 195) and those that experienced childbirth without maternal death (n = 384) in rural China. All the households were interviewed after the event occurred and were followed up 12 months later. Structural equation modeling was used to test the relationship model, utilizing income and expenditure per capita in the following year after the event as the main outcome variables, maternal death as the predictor, and direct costs, the amount of money offset by positive and negative coping strategies, whether the husband remarried, and whether the newborn was alive as the mediators.

**Results:**

In the following year after the event, the path analysis revealed a direct effect from maternal death to lower income per capita (standardized coefficient = -0.43, *p* = 0.041) and to lower expenditure per capita (standardized coefficient = -0.51, *p*<0.001). A significant indirect effect was found from maternal death to lower income and expenditure per capita mediated by the influencing factors of higher direct costs, less money from positive coping methods, more money from negative coping, and the survival of the newborn.

**Conclusion:**

This study analyzed the direct and indirect effects of maternal death on a household economy. The results provided evidence for better understanding the mechanism of how this event affects a household economy and provided a reference for social welfare policies to target the most vulnerable households that have suffered from maternal deaths.

## Introduction

According to the latest data issued by the United Nations, approximately 287,000 cases of maternal death occurred worldwide in 2010 [1]. As we approach 2015—the deadline for achieving Millennium Development Goal 5A (to reduce the maternal mortality ratio by three-quarters), the maternal mortality ratio has only declined by 47% from 1990, which is of concern to the international community [1].

Lack of financing is a key constraint for the reduction of maternal mortality [2,3]. Compared to those of HIV/AIDS, malaria and TB (tuberculosis) in recent years, the funding levels for maternal, newborn, and child survival services have decreased significantly relative to the demand of services. The trend of increasing aid volume on maternal, newborn, and child survival was replaced by a slight decrease of 0.5% [3]. As one of the countdown priority countries, China has received official development assistance, but this aid has decreasing by 0.5% annually since 2003[3].

The worldwide reduction of aid is partly because reproductive health advocates have not been as effective as advocates of other diseases, particularly HIV/AIDS, malaria and TB, in demonstrating the fact that failure to address the reproductive health problems of poor women can undermine poverty reduction [4]. One of the difficulties in advocating for reproductive health is that maternal death is a relatively rare demographic event, and it is difficult to enroll sufficient cases [5]. We conducted a systematic search on the electronic databases of Pubmed, Embase and Popline and on the home pages of major international organizations for published literature. We only identified one study that was performed to estimate the immediate costs of maternal death, which was from our baseline data [6]. Moreover, little is known about how such an immediate exposure or how a family copes with such an exposure affects their household economy in the long-term because no study has previously followed households to examine the effects of maternal death. Recently, there are emerging studies investigating the costs of emergency obstetric care [7,8], which have been found to be a useful complement to investigations of maternal mortality. Two of these studies have investigated the forward effects of high costs related to experienced emergency obstetric care [7,8]. However, whether the women survived might be crucial for a household economy because households may be challenged by maternal death with its double jeopardy of losing its “home economist” and diminished productivity [9,10].

**Fig 1:**
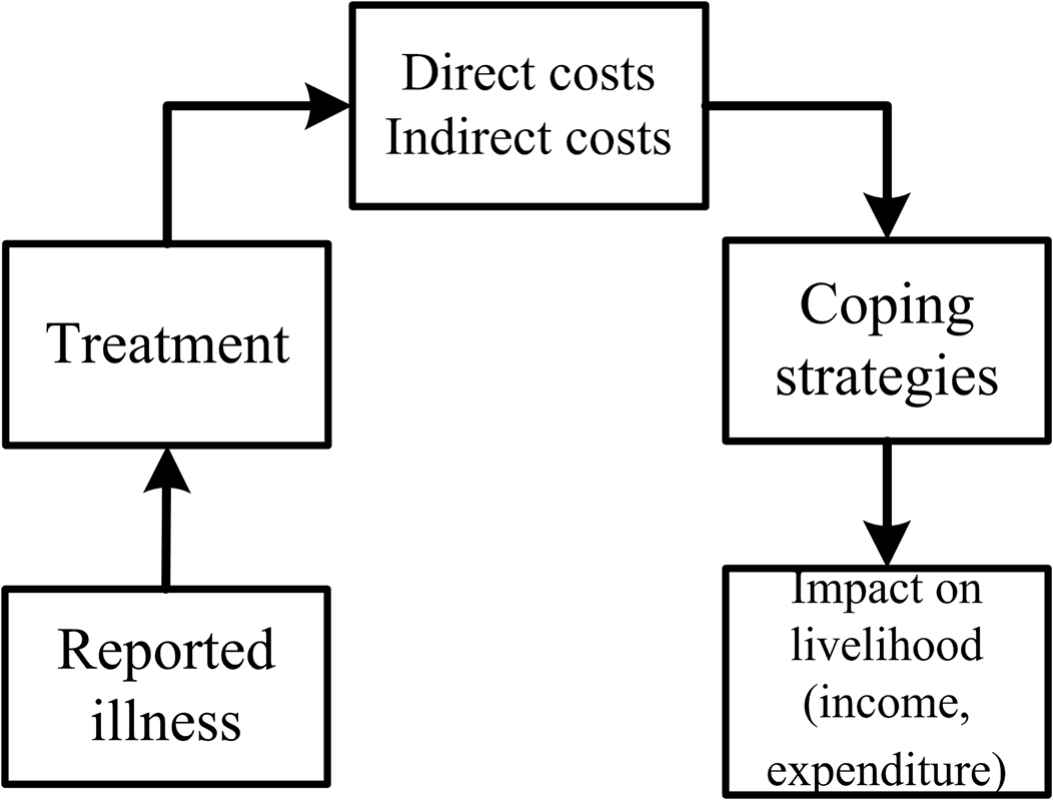
Conceptual framework for analyzing the economic burden of illness on households by Russell [11].

Based on scientific evidence from published literatures of economic studies of other illness, a conceptual framework was derived by Russell (Fig 1) [11]. The conceptual framework deduced the path from illness to the household economy. According to the framework, after a “reported illness”, the individual or household decides whether to seek treatment or not and what treatment to take. The treatment triggers illness costs, including both direct and indirect costs. Direct costs refer to all the household expenditure linked to seeking treatment. Indirect costs refer to the loss of productivity because of the illness. In response to the illness costs, the household must cope with both the direct and indirect costs. Coping strategies include coping with direct costs (borrowing, taking loans, etc.) and coping with indirect costs (household composition alteration, etc.). The immediate costs and coping strategies will affect the maintenance and generation of the household's asset portfolio. Based on this framework, we explored the economic effects of maternal death on households using data from our cohort study of maternal death in Chinese rural households.

This cohort study was initiated in 2009 among households that had experienced childbirth with and without maternal death in rural areas of three provinces in China. We have previously reported the baseline study findings (cost-of-illness of maternal death) [6]. The median of the immediate and non-reimbursed direct economic burden of the households that had suffered from maternal death was 37.0% of the household's annual income, which was approximately 4 times greater than the threshold for an expense being considered catastrophic. In addition, the high direct economic burden was coupled with fewer assets, reduced savings and increased debt, which were likely to affect all surviving family members during the following period. Examining only the immediate economic effect is insufficient for fully understanding the effect of maternal death on a household economy. Therefore, we followed up these households 12 months later to track the long-term effects and to identify factors influencing the economic effect. The aim of this study was to explore the relationships among maternal death, household economic status after the event, and potential influencing factors.

## Methods

This was a prospective cohort study. The baseline survey data were collected from June 2009 through October 2011, and the follow-up surveys were conducted one year after the baseline survey. The investigation was administered in rural areas of Hebei, Henan, and Yunnan provinces of China, which were purposively selected to represent settings with low, moderate, and high MMRs (maternal mortality ratio), with consideration of certain geographic areas, population sizes, and transportation routes.

The study matched 195 households that had experienced maternal death with 384 households of childbirth without maternal death. The study protocol and data collection instruments were approved by the ethical reviews boards of Peking University and the World Health Organization. Signed informed consent statements were obtained prior to any interviews. All the interviews were entirely voluntary (no incentives were provided).

### Participants

Before the study began, we calculated a minimum sample size of 132 households of maternal death and 264 comparison households to achieve a power of 0.8. The minimum sample size was inflated to 195 households of maternal death and 390 comparisons to compensate for an expected attrition rate (because of family dissolution or movement out of the study sites), general non-response or non-compliance, and desired sub-group analyses. The sample size was distributed to each of the selected provinces according to their MMRs in 2008. The study was open to all cases of maternal death during the study period until the sample size was achieved. To be considered for inclusion in this study, households that had experienced maternal death within three months were first identified by the County Maternal and Child Health Office in each of the selected provinces according to the inclusion criteria listed below; these offices routinely reported each case of maternal death within their administrative areas through a system including village level, township level, county level, and provincial level, no matter whether the deceased women gave birth in the hospital or at home.

### Inclusion criteria for the households with maternal death

- Having maternal death within 3 months of the interview; maternal death was defined as the death of a woman while pregnant (≥28 weeks gestational age) or within 42 days of the termination of pregnancy but not for accidental causes unrelated to the pregnancy.

Each affected household that had experienced maternal death was matched with two comparison households according to the inclusion and exclusion criteria as follows.

### Matching criteria for the households of childbirth without maternal death

- Having childbirth within 3 months of the interview;
- Living in the same administrative village;
- Similar economic status evaluated by the administrative village cadres (i.e., rich, moderate, poor);
- The household type was the same as that of the affected family before maternal death (i.e., nuclear or extended; with or without older children) and;
- The ethnicity of the head of the family was identical to that of the maternal death family, if possible.

**Fig 2:**
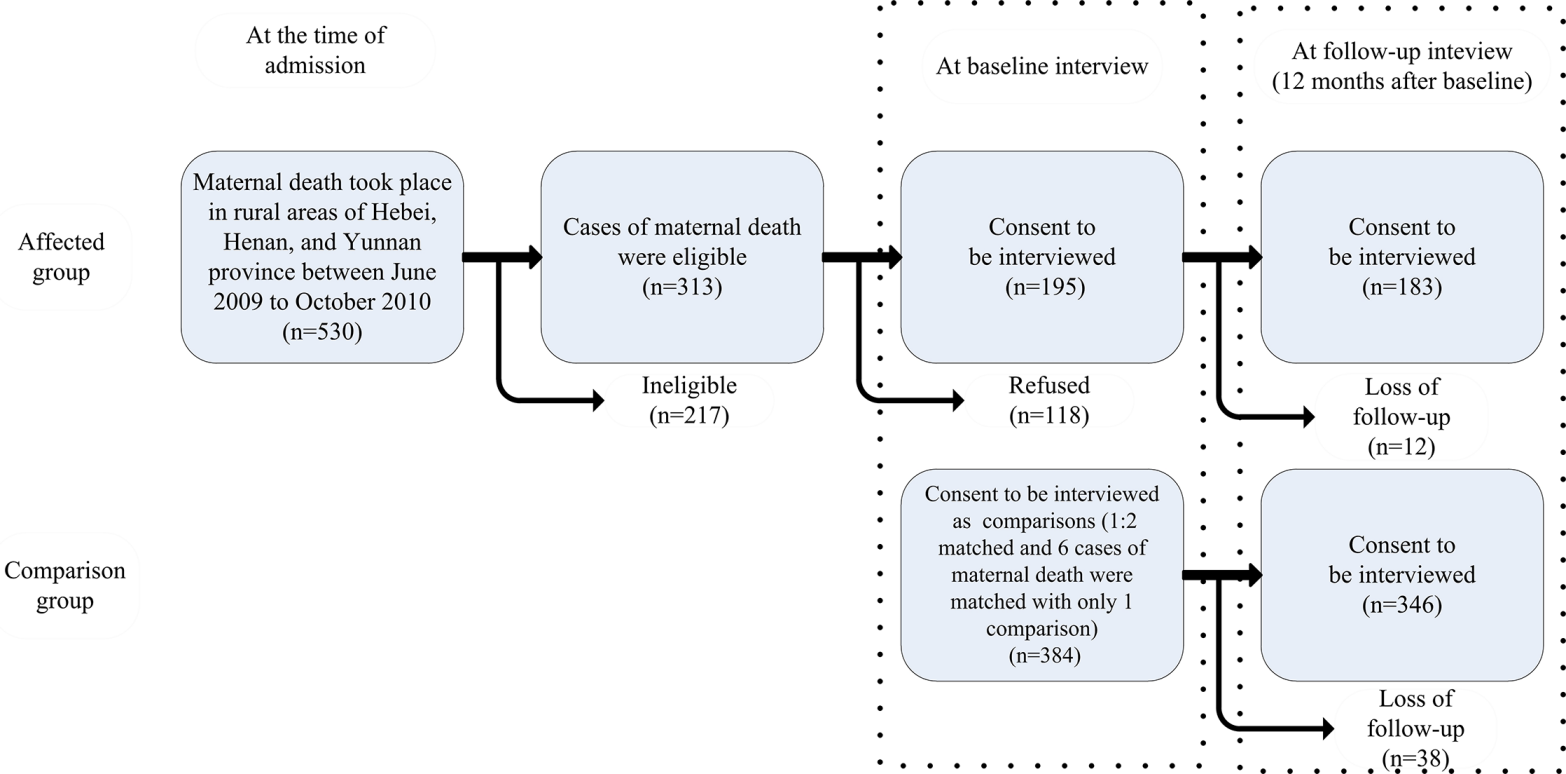
Flowchart of the subjects in the two groups in the study.

There were 530 cases of maternal death that occurred in the study area. Of these cases, 40.9% were ineligible or did not meet the inclusion criteria (217 out of 530), and an additional 22.3% of the cases (118 out of 530) were eligible but refused to be interviewed. Finally, 195 households agreed to be interviewed. We matched these affected households with 384 comparisons according to the inclusion criteria (6 affected households were only matched with one comparison in the remote and under-populated areas). In the follow-up survey that was conducted one year after the baseline survey, 183 affected households and 346 comparisons were followed up. The attrition rate was 8.6% (50/579) (Fig 2).

### Measures

#### Dependent variables

The two dependent variables were continuous variables that represented the annual income and expenditure per capita in the household. They were derived by summing the 32 component items of income and 48 items of expenditure in detail to generate the total annual household income and expenditure before and after maternal death/childbirth; then the annual household income and expenditure were divided by household size (number of family members). In the following description, we used “baseline income” and “baseline expenditure” to represent the annual income and expenditure per capita in the year before maternal death/childbirth, and we used “follow-up income” and “follow-up expenditure” to represent the annual income and expenditure per capita in the year after maternal death/childbirth.

#### Independent variable

We defined whether the household had experienced maternal death as the independent variable to assess its direct and indirect effects on the household economy, which was represented by the indicators “follow-up income” and “follow-up expenditure”.

#### Mediators

To explore the pathways between maternal death and “follow-up income” and “follow-up expenditure”, we examined five mediators, including direct costs related to maternal death/childbirth (“direct costs”), two sources of financial support used to offset the direct costs (“positive coping” and “negative coping”), whether the husband remarried after the maternal death/childbirth (“husband remarried”), and whether the newborn was alive until the follow-up interview (“newborn was alive”). In the following model, affected households suffered from maternal death was defined as “1”, comparison households was defined as “0”. Household households with husband remarried during the following year was defined as “1”, while households without remarriage was defined as “0”. Households with newborn did not survived until follow-up interview was defined as “0”, and other households with infants was defined as “1”.

The direct costs included hospitalization and emergency costs, transportation costs, extra costs, and funeral costs, which were listed as four items in the questionnaire in our baseline study [6]. “Positive coping” and “negative coping” were two major types of financial support that were used to offset the direct costs. “Positive coping” refers to financial supports from insurance reimbursement, hospital compensation and government subsidies. “Negative coping” refers to financial sources from inside the household such as selling assets, mobilizing available cash or savings and borrowing money/taking loans.

### Statistical analyses

The database was developed using Epidata 3.1 software. The statistical analyses were conducted with SPSS ver. 15.0 software for Windows (Statistical Package for Social Science, Inc., Chicago, USA), and Mplus [12]. First, we conducted Chi-square and t-tests to examine the bivariate relationships between maternal death and the mediators (“direct costs”, “positive coping”, “negative coping”, “husband remarried” and “newborn was alive”) and between the mediators and dependents (“follow-up income” and “follow-up expenditure”) using SPSS. Next, MPlus was employed for a path analysis. This technique was applied to test the conceptual pathway of the potential factors. Income, expenditure, and the amount of each type of coping had positively skewed distributions and were logarithmically transformed before the statistical analyses. As “maternal death”, “husband remarried”, and “newborn was alive” were dichotomous variables, WLSMV (Weighted Least Squares with Adjusted Mean and Variance) parameterizaton method was utilized.

## Results

### General characteristics

The general characteristics of the participants demonstrated a successful matching for the two study groups (Table 1). The two groups did not differ in household size (*p =* 0.884) and in the proportion of nuclear families (*p =* 0.809). Women in the two groups did not differ in proportion of minorities (*p =* 0.255), the proportion of primiparas (*p =* 0.263), the proportion of peasants or housewives (*p =* 0.673), and education duration (*p =* 0.277). Women in the affected group tended to be older than those in the comparison group (*p<*0.001). Therefore, maternal age was selected as one of the covariates to be controlled for within the following structural equation modeling.

**Table 1:** Comparison of the background characteristics between the two groups.

Variable	Affected group (N = 183)	Comparison group (N = 346)	Statistics	*p*-Value
*Household characteristics*				
Household size before maternal death/childbirth, mean (SD)	4.5 (1.4)	4.5 (1.6)	3.074	*0*.*884* ^*a*^
% nuclear family	32.2	31.2	0.058	*0*.*809* ^*b*^
Baseline income per capita				
Baseline expenditure per capita				
*Women’s characteristics*				
Women’s age, median (min-max)	30.0 (19.0–46.0)	27.0 (18.0–43.0)	4.623	*<0*.*001* ^*c*^
% minorities	25.1	20.8	1.293	*0*.*255* ^*b*^
% primiparas	36.6	41.6	1.251	*0*.*263* ^*b*^
% peasants or housewives	86.9	85.5	0.178	*0*.*673* ^*b*^
% with less than 9 years of education	94.0	91.3	1.184	*0*.*277* ^*b*^

^a^ t-test

^b^ Chi-square test

^c^ rank-sum test

### Bivariate analyses

Compared to the households without maternal death, the affected households were more likely to have reported “husband remarried”, higher amount of “direct costs”, and more money offset by both “positive coping” and “negative coping” but were less likely to have reported that the “newborn was alive” (Table 2). In addition, there were significant differences between the two groups in “baseline income” and “baseline expenditure”. Therefore, we selected “baseline income” and “baseline expenditure” as covariates in the following structural equation modeling.

**Table 2:** Bivariate relationships with maternal death.

Variable ^a^	Affected group (n = 183), freq	Comparison group (n = 346), freq	χ^2^	*p*-Value
Husband remarried	26	0	51.70	*<0*.*001*
Newborn was alive	102	346	178.21	*<0*.*001*
	Mean (SD)	Mean (SD)	t-test	*p-*Value
Direct costs	9.76 (0.99)	7.21 (1.54)	20.23	*<0*.*001*
“Positive coping”	0.87(1.14)	-0.46(0.48)	14.98	*<0*.*001*
*C1 (Hospital compensation)*	4.63 (5.26)	0.00 (0.00)	11.89	*<0*.*001*
*C2 (Government cash assistance)*	0.84 (2.15)	0.03 (0.40)	5.02	*<0*.*001*
*C3 (Medical insurance reimbursement)*	2.14 (3.44)	4.73 (2.87)	-8.69	*<0*.*001*
“Negative coping”	0.65(1.12)	-0.34(0.73)	10.91	*<0*.*001*
*C4 (Selling assets)*	0.59 (2.09)	0.04 (0.52)	3.52	*0*.*001*
*C5 (Borrowing money or taking loans)*	6.93 (4.21)	1.96 (3.42)	13.76	*<0*.*001*
*C6 (Mobilizing available cash or savings)*	3.35 (4.13)	2.53 (3.38)	2.30	*0*.*022*
Baseline income ^b^	8.11 (0.76)	8.40 (0.78)	-4.04	*<0*.*001*
Baseline expenditure ^c^	8.39 (0.85)	8.54 (0.78)	-2.07	*0*.*039*
Follow-up income ^d^	8.21 (0.78)	8.41 (0.66)	-3.02	*0*.*003*
Follow-up expenditure ^e^	8.13 (0.76)	8.15 (0.64)	-0.34	*0*.*734*

^a^ Income, expenditure, and the amount of each type of coping had positively skewed distributions and were logarithmically transformed

^b^ Annual income at the individual level before maternal death/childbirth

^c^ Annual expenditure at the individual level before maternal death/childbirth

^d^ Annual income at the individual level after maternal death/childbirth

^e^ Annual expenditure at the individual level after maternal death/childbirth

As shown in Table 3, there were significant effects of “husband remarried”, “baseline income”, and “baseline expenditure” on both “follow-up income” and “follow-up expenditure”. The amount of money offset by both “positive coping” and “negative coping” had significant effects on “follow-up income”. Higher “direct costs” was significantly related to higher “follow-up expenditure”.

**Table 3:** Bivariate relationships with “follow-up income” and “follow-up expenditure”.

Variable ^a^	Correlation with follow-up income ^d^		Correlation with follow-up expenditure ^e^	
	β^f^	*p*-value	β	*p*-value
Husband remarried	0.10	*0*.*021*	0.19	*<0*.*001*
Newborn was alive	-0.04	*0*.*038*	0.01	*0*.*824*
Direct costs	0.06	*0*.*182*	0.14	*0*.*001*
“Positive coping”	-0.14	*0*.*002*	0.02	*0*.*625*
*C1 (Hospital compensation)*	-0.09	*0*.*048*	0.06	*0*.*195*
*C2 (Government cash assistance)*	-0.06	*0*.*146*	-0.05	*0*.*259*
*C3 (Medical insurance reimbursement)*	0.12	*0*.*007*	0.07	*0*.*103*
“Negative coping”	-0.17	*<0*.*001*	-0.08	*0*.*057*
*C4 (Selling assets)*	-0.09	*0*.*049*	-0.07	*0*.*126*
*C5 (Borrowing money or taking loans)*	-0.20	*<0*.*001*	-0.07	*0*.*125*
*C6 (Mobilizing available cash or savings)*	-0.02	*0*.*707*	-0.02	*0*.*576*
Baseline income ^b^	0.61	*<0*.*001*	0.47	*<0*.*001*
Baseline expenditure ^c^	0.45	*<0*.*001*	0.56	*<0*.*001*

^a^ Income, expenditure, and the amount of each type of coping had positively skewed distributions and were logarithmically transformed

^b^ Annual income at the individual level before maternal death/childbirth

^c^ Annual expenditure at the individual level before maternal death/childbirth

^d^ Annual income at the individual level after maternal death/childbirth

^e^ Annual expenditure at the individual level after maternal death/childbirth

^f^ Regression coefficient estimate

### Structural Equation Modeling

**Fig 3:**
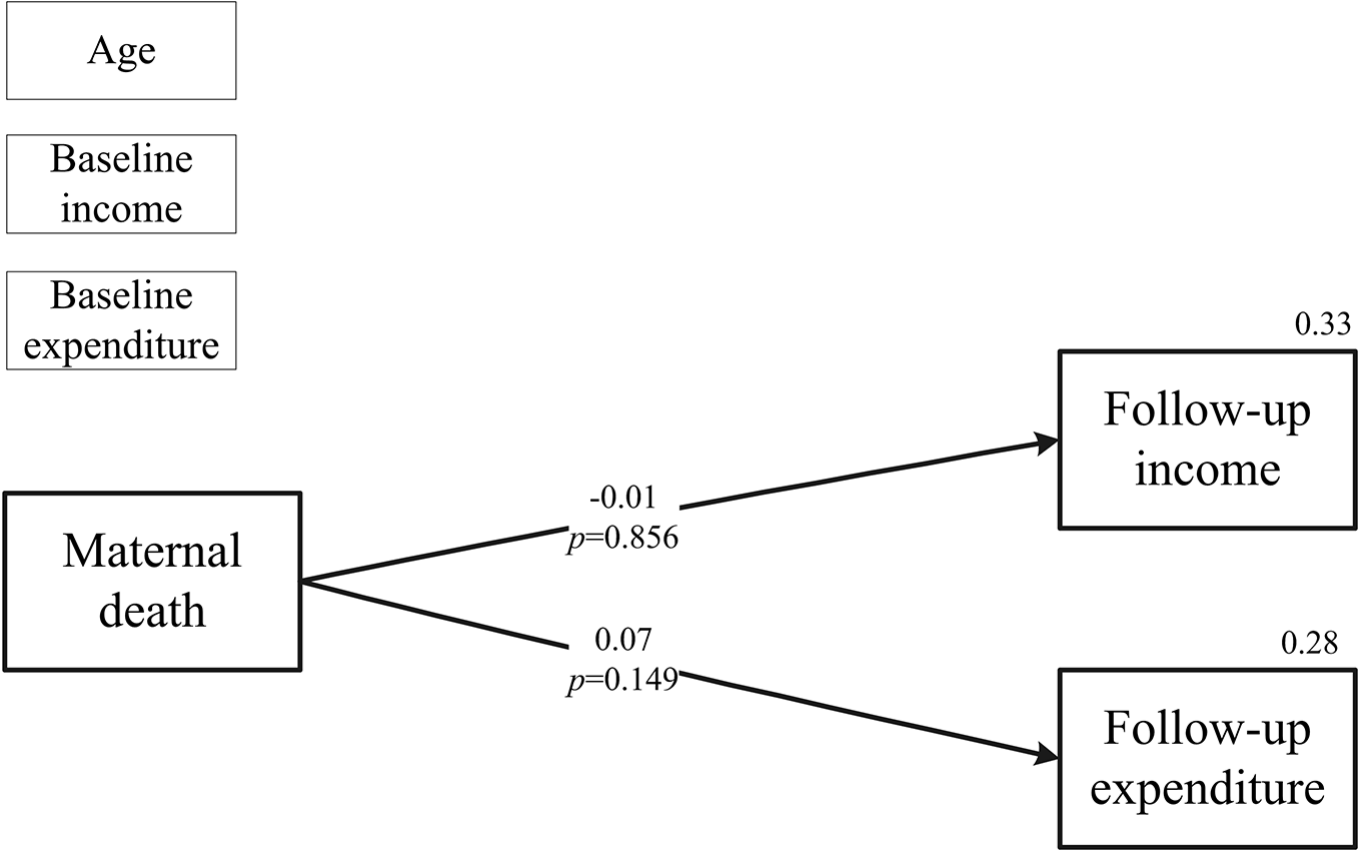
Relationships between maternal death on follow-up income and maternal death and follow-up expenditure.

The pathway from maternal death to household economy was then analyzed by SEM modeling. Model 1 focused on the total effect of maternal death on “follow-up income/expenditure”. Fig 3 showed that the event of maternal death was not significantly related to both “follow-up income” and “follow-up expenditure”. The model fit indices showed that the Chi-square/df = 36.307/2, the CFI (comparative fit index) was 0.938, the TLI (Tucker Lewis Index) was 0.721, and the SRMR (standardized root mean square residual) was 0.099.

**Fig 4:**
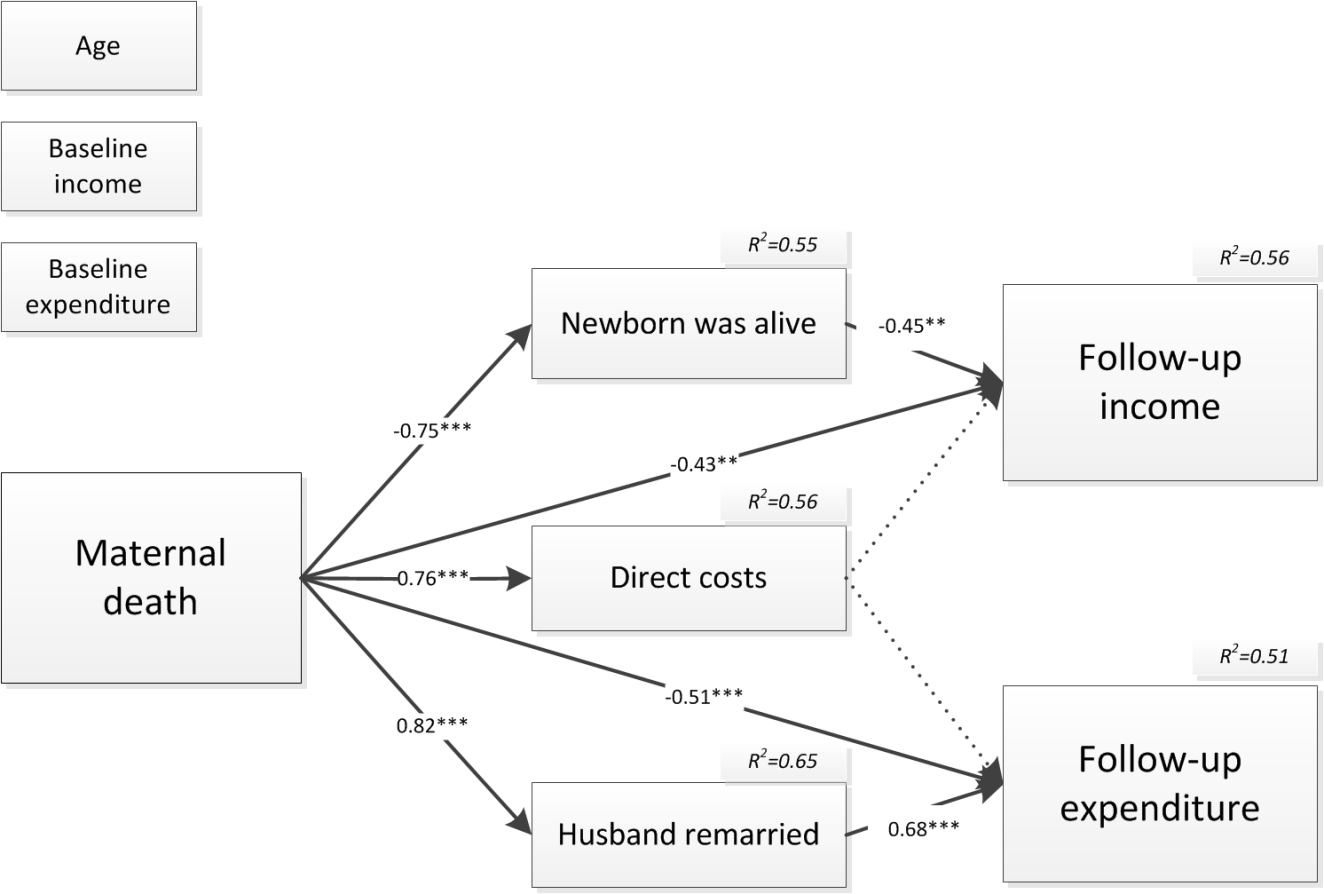
Path model from maternal death to follow-up income and expenditure via mediators with standardized coefficients. * at 10% significance, ** at 5% significance, *** at 1% significance

**Fig 5:**
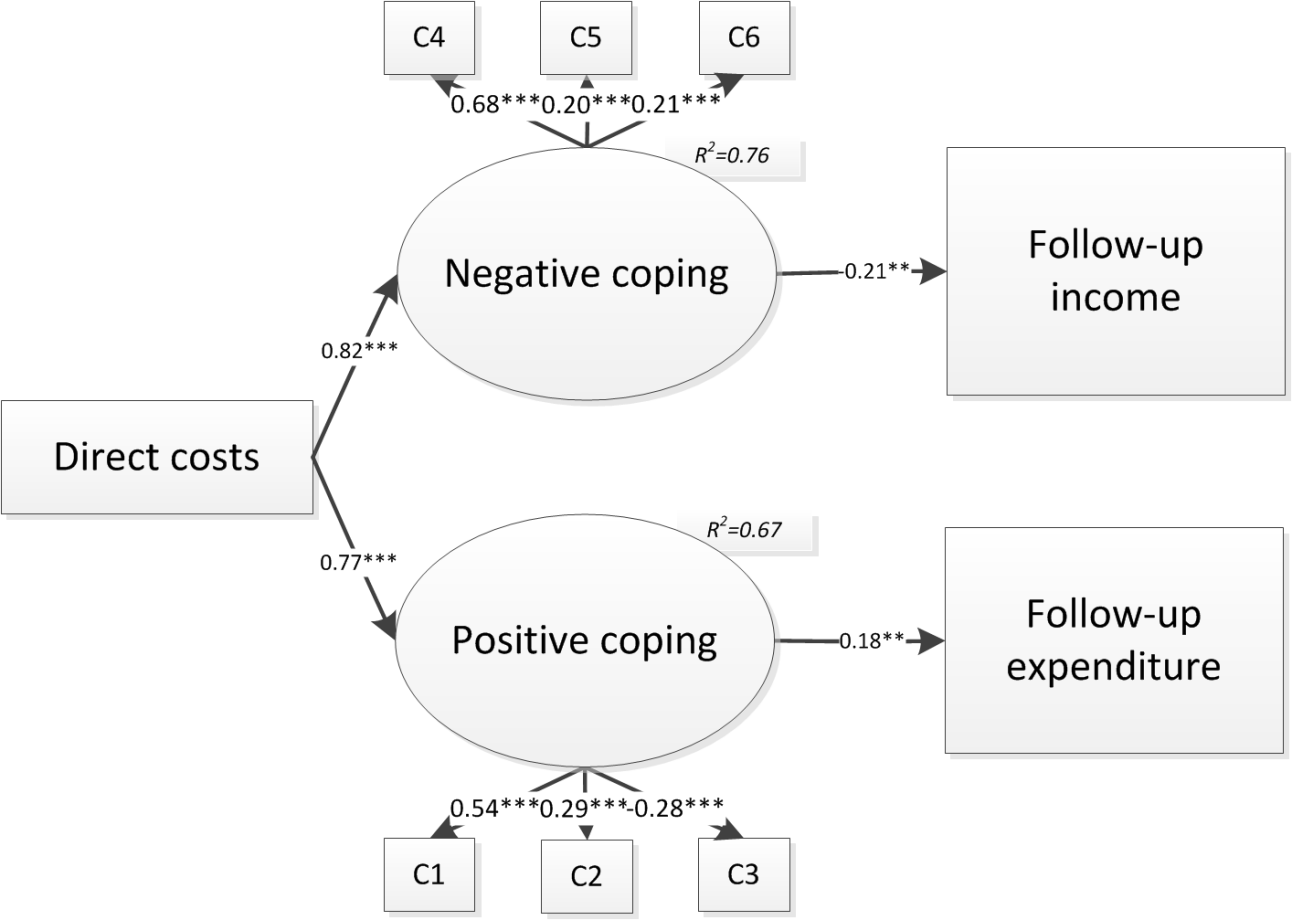
Path model from maternal death to follow-up income and expenditure via mediators with standardized coefficients. * at 10% significance, ** at 5% significance, *** at 1% significance

We further analyzed the relationship between maternal death and “follow-up income/expenditure” when “husband remarried”, “newborn was alive”, “direct costs”, “positive coping”, and “negative coping” were added. The structural model explained 56% and 51% of the total variance in “follow-up income” and “follow-up expenditure”, respectively. Figs 4 and 5 shows the factor loadings and the R squares in the model. The model fit indices showed that the Chi-square/df = 134.56/66, the CFI was 0.963, the TLI was 0.941, the RMSEA (root mean square error of approximation) was 0.044 (90% CI: 0.034–0.055), and the WRMR (weighted root mean residual) was 0.899, indicating an excellent model fit; additionally, all factors in the model had significant effects on the dependents of both “follow-up income/expenditure”.

Fig 4 showed that when controlling for the age of the woman, “baseline income” and “baseline expenditure”, maternal death directly predicted lower “follow-up income” and “follow-up expenditure” (all *p*<0.05).

Besides, suffering from maternal death had a significant positive association with “husband remarried” (standardized coefficient = 0.82, *p<*0.001), and “husband remarried” further increased “follow-up expenditure” significantly (standardized coefficient = 0.68, *p<*0.001).

Less newborns from affected households with maternal death were alive compared with comparison households (standardized coefficient = -0.75, *p<*0.001), and there was a negative relationship between “newborn was alive” and “follow-up income”(standardized coefficient = -0.45, *p = 0*.*003*).

Maternal death significantly increased the “direct costs” immediately after maternal death associated with both medical and funeral expenses(standardized coefficient = 0.76, *p<*0.001). “Direct costs” associated with different coping methods which had further impact on both follow-up income and follow-up expenditure (See Fig 5).

As Fig 5 showed, higher “direct costs”, higher “direct costs” were significantly associated with a higher amount of both “negative coping” (standardized coefficient = 0.82, *p<*0.001) and “positive coping” (standardized coefficient = 0.77, *p<*0.001). Interestingly, “negative coping” and “positive coping” had different impact on “follow-up income” and “follow-up expenditure”.

The amount of “negative coping” was negatively associated with “follow-up income” (standardized coefficient = -0.21, *p =* 0.032), while the amount of “positive coping” was positively associated with “follow-up expenditure” (standardized coefficient = 0.18, *p =* 0.037).

Table 4 indicates the direct, indirect and total effects of maternal death on “follow-up income and expenditure”. According to the results, maternal death had a significant indirect relationship with “follow-up income” and “follow-up expenditure” through its effect on “direct costs”, the amount of money covered by “positive coping” and “negative coping”, “newborn was alive” and “husband remarried” during the following year.

**Table 4:** Direct, indirect, and total effects of maternal death on follow-up income and expenditure.

	Model	Variables	Standardized estimate	Standard error	P-value
Follow-up income ^a^	Direct effect	Maternal death → follow-up income	-0.43	-0.20	0.041
	Indirect effect	Maternal death → direct cost → positive coping → follow-up income	-0.07	0.05	0.142
		Maternal death → direct cost → negative coping → follow-up income	-0.13	0.06	0.040
		Maternal death → newborn alive → follow-up income	0.33	0.12	0.005
		Maternal death → husband remarried → follow-up income	0.30	0.19	0.108
	Total effect	Direct + indirect	-0.01	0.04	0.856
Follow-up expenditure ^b^	Direct effect	Maternal death → follow-up expenditure	-0.51	0.13	<0.001
	Indirect effect	Maternal death → direct cost → positive coping → follow-up expenditure	0.10	0.05	0.027
		Maternal death → direct cost → negative coping → follow-up expenditure	-0.05	0.07	0.412
		Maternal death → newborn alive → follow-up expenditure	-0.03	0.09	0.741
		Maternal death → husband remarried → follow-up expenditure	0.56	0.11	<0.001
	Total effect	Direct + indirect	0.07	0.05	0.149

^a^ Annual income at the individual level after maternal death/childbirth

^b^ Annual expenditure at the individual level after maternal death/childbirth

Maternal death had indirect effects from high direct costs caused by maternal death on “follow-up income” via “negative coping” (standardized estimate effect = −0.13, *standard error* = 0.06, *p =* 0.040) and its indirect effects on “follow-up expenditure” via “positive coping” (standardized estimate = 0.10, *standard error* = 0.05, *p =* 0.027). Interestingly, we found no significant paths from “direct costs” to “follow-up income” via “positive coping” or from “direct costs” to “follow-up expenditure” via “negative coping”, indicating that there are likely different mechanisms of how “direct costs” affect household income and expenditure.

## Discussion

This study quantifies the direct and indirect effects of maternal death on the household economy in the following year. To our knowledge, this is the first evidence from a prospective cohort study. The main finding was that maternal death is associated with both a lower income and expenditure per capita in the following year of the event via direct costs, coping strategies, newborn status, and husband marriage status.

The quantitative results showed that the total effect of maternal death on both follow-up income and expenditure per capita was not statistically significant. Conversely, according to our previous study [13], there was a nearly four times higher risk of the affected households reporting “household economy became worse” than in the comparison households in the following year after the maternal death. The reason of the insignificant total effect of maternal death on income or expenditure was that the newborn mortality accompanied by the maternal death reduced the household size, which further increased the income or expenditure per capita. Thus, the deceased women have a dual effect on the household economy by changing the household composition. Therefore, separating the direct and indirect effects may help us better understand the mechanism of how maternal death affects the household economy and provide references for social welfare policies to target the most vulnerable households suffering from this event.

According to Russell’s conceptual framework [11], households utilized social networks to cope with both direct costs and indirect costs that were incurred by the maternal death and relevant treatment. In our study, a maternal death affects the household economy in two major ways: high medical/funeral related costs (direct costs) and reduced productivity (indirect costs). Each type of effect triggers relevant coping strategies in the household, including economic coping strategies (e.g., borrowing or selling assets) and household management coping strategies (e.g., reduced income-generated work to raise the surviving newborn, husband remarried). Additionally, raising newborns and the remarriage triggers a large amount of expenditure. Thus, different coping strategies themselves further affect the household economy in different ways. Analyzing the influence of each mediator on both income and expenditure is critical to estimate the direct and indirect effects of maternal death on the household economy.

In this study, we found that money from “negative coping” reduced the “follow-up income” in the affected households. Although certain coping methods would cover the gap between high immediate costs and household wealth, it diminished both current welfare and future prospects of the households by selling assets, borrowing money or taking loans, and mobilizing available cash or savings. Negative coping reduced the household economy “buffer” and lessened household productive properties, which made it impossible for the family to increase or maintain the household income.

Meanwhile, money from “positive coping” increased “follow-up expenditure” in the affected households. The bivariate analysis showed that “positive coping” was higher in the affected households; however, money from medical insurance reimbursements received by these households was less than the comparison households (2.14 vs. 4.73, p<0.001). Although the affected households received higher cash assistance from the government, it only covered a small proportion of “positive coping” and was less than an average of US $15 in each household. Increased financial relief from the government could contribute to the poverty alleviation of households suffering from maternal death and may to some extent prevent informal financial protection from being utilized by the affected households, such as illegally receiving compensation for hospital mal-practice. However, those affected households that received less money as “positive coping” had to reduce their living consumption to sustain the household economy. Therefore, financial assistance would protect the living conditions of the surviving households.

Newborns have dual effects on “follow-up income” and “follow-up expenditure”. First, family members of the deceased women must reduce their income-generated work to raise the surviving newborn or change the income source from outside employment to family production, both of which result in an income loss. Secondly, those surviving newborns that lost their mothers require exclusive formula feeding, which is far more expensive than breast feeding. In our study, 81 cases of newborns (56.3%) in the affected households and 0 cases (100.0%) in the comparison households were dead at the follow-up interview, including 46 cases from women who died before delivery, 21 cases of stillbirth, and 14 cases of newborns who died during the period between the maternal death and the follow-up interview. Although we separated the expenditure of raising surviving newborns from the household annual expenditure in this study to increase the comparability between the “baseline expenditure” and “follow-up expenditure”, the newborn relevant expenditure could not be ignored. The median expenditure for raising the surviving newborn was US $1007, which was more than 2 times higher compared to that in the comparison households (US $484).

In rural China, marriage/remarriage costs an enormous amount of money for the wedding ceremony, and a large amount of money is given to the bride’s family as cash gifts. In addition, the groom’s family also receives a large amount of money from relatives and friends in the wedding ceremony. The above mentioned cultural traditions of marriage have affected both follow-up income and follow-up expenditure. Additionally, remarried wives could be a good substitute for the labor loss caused by the deceased women, which increased the household income as well. In this study, husbands from 26 affected households remarried in the year following the maternal death, and the mean expenditure for remarriage of the husbands in affected group (26 cases) was as high as US$ 4667.

This study has several implications for health policies. First, it quantified the effect of maternal death and several mediators on household economy, which could help the government target the most vulnerable households: those that paid high direct costs, obtained low medical reimbursement and government cash assistance, were in debt, sold assets and were left with a surviving newborn. Financial protection against the high direct costs of emergency obstetric care through tax- or insurance-based financing systems would be crucial in protecting households from catastrophic payment [8]. Positive coping strategies, which included financial supports from insurance reimbursement, hospital compensation and government subsidies, were several types of financial protection households could receive in this study. Notably, the medical insurance reimbursement received in the affected households was significantly lower than that in the comparison households. Further studies should explore the reason why the affected households obtained less reimbursement from emergency obstetric care. The results showed that the cash assistance from the government only covered a small portion of direct costs [6]. Certain policies or programs should focus on these vulnerable households suffering from maternal death.

## Conclusions

The study analyzed the direct and indirect effects of maternal death on the household economy. The results provided evidence for better understanding the mechanisms of how such an event affects a household economy and provided reference for social welfare policies to target the most vulnerable households suffering from maternal deaths. Adverse direct effects of maternal death on the household economy can impoverish households or push them further into poverty. An increase of funding for maternal mortality prevention could contribute to a poverty reduction.

## Supporting Information

S1 Dataset (RAR)
